# Listeners are biased towards voices of young speakers and female speakers when discriminating voices

**DOI:** 10.1186/s41235-025-00636-3

**Published:** 2025-06-07

**Authors:** Valeriia Vyshnevetska, Nathalie Giroud, Meike Ramon, Volker Dellwo

**Affiliations:** 1 Linguistic Research Infrastructure, University of Zurich, Andreasstrasse 15, 8050 Zurich, Switzerland; 2 Department of Computational Linguistics, University of Zurich, Zurich, Switzerland; 3 Applied Face Cognition Lab, Business School, Bern University of Applied Sciences, Bern, Switzerland; 4 AIR – Association for Independent Research, Zurich, Switzerland

**Keywords:** Speaker recognition, Own-age effect, Response bias

## Abstract

In face processing, an own-age recognition advantage has frequently been reported whereby observers are better at recognizing faces of their own compared to other age groups. We wanted to know whether own-age effects exist in voice recognition. Two listener groups, younger adults (*n* = 42, 19–35 years, 21 males) and older adults (*n* = 32, 65–83 years, 14 males), completed a speaker discrimination task (same/different speakers), which included younger and older adult speakers of both sexes. Results revealed no interaction of the factors speaker and listener age and speaker and listener sex on listeners’ sensitivity (*d*′). Main effects were significant for listener age (young adult listeners exhibited higher sensitivity than the older adult listeners) and speaker sex (listeners’ sensitivity was higher for male compared to female voices). Crucially, response bias (*c*) revealed that listeners had a significantly higher ‘same’ bias when hearing younger speakers and female speakers. Our findings have implications for theories of voice identity processing and forensic contexts requiring discrimination of speakers’ identity, e.g. earwitnesses telling apart younger and female speakers.

## Significance statement

Recently, ‘fake police officer’ crimes were reported in many countries. In these scenarios, older individuals are contacted by younger persons pretending to be police officials and attempting to deceive older adults of their valuables. Crucially, the younger individuals do not represent official state security but are part of the arranged scam. What is more, the so-called deepfake voices are gaining popularity, by which novel speech utterances can be generated with an individual’s voice using artificial intelligence techniques. Audio deepfakes have led to crimes whereby victims are tricked to believe that one of their family members or close friends is in urgent need of financial help, and in such scenarios, fraudsters’ and victims’ age may differ drastically. Thus, an earwitness at court might have to judge whether the voice of a suspect from a strongly different age group belongs to a speaker they spoke to on the telephone during the voice crime. However, it is unclear whether listeners are better at recognizing voices of their own age compared to other ages. This study showed that when hearing younger speaker pairs and female speaker pairs, listeners are significantly biased to saying that both excerpts stem from the same speaker. In voice crime cases discussed earlier, this could imply that earwitnesses might find it more challenging to discriminate younger speakers and female speakers, especially if the audio quality is poor.

## Introduction

Voice recognition is a seemingly effortless everyday task which nevertheless can be affected by many speaker-, listener-, and channel-related factors such as listener’s degree of familiarity with the voice (Hollien et al., 1982; Schmidt‐Nielsen & Stern, 1985), voice distinctiveness (Papcun et al., 1989), duration of exposure to a voice (Clifford, 1980; Foulkes & Barron, 2000; McGehee, 1937), the amount of time elapsed between exposure and test (Clifford, 1980), as well as communication channel quality (McDougall et al., 2015; Nolan et al., 2013; Rathborn et al., 1981), to name just a few (for a review of different factors, see Jessen (2008); McDougall et al. (2015)). Listener’s age has also been shown to affect various voice perception tasks, with younger adult listeners outperforming older adult listeners (Best et al., 2018; Goy et al., 2016; Kausler & Puckett, 1981; Moyse et al., 2014; Schvartz & Chatterjee, 2012; Zaltz & Kishon-Rabin, 2022). However, it remains unclear whether younger adults outperform older adults in recognizing voices of all ages or only of voices of their own-age group, a phenomenon referred to as own-group advantage in other contexts, predominantly face recognition (see below). This study explores own-age advantages in voice discrimination.

Recently, voice crime scenarios became widespread whereby older adults are contacted on the phone by young individuals pretending to be police officers and informed about criminal activity in the nearby area (Action Fraud, 2015; Hadfield, 2024). As a security measure, older adult persons are instructed to hand over valuables they possess at home to the fictitious police officers or provide access to their bank account. Crucially, the young callers do not represent any official state security but are part of an arranged scam by which older adults get deceived of their possessions. Such scenario is known as the ‘fake police officer’ crime, and numerous variants of this type of voice crimes are frequently reported nowadays from many countries such as the UK (Ball, 2023; Loffreda, 2023), Germany (Schumacher, 2019) or Switzerland (Der Landbote, 2023; Swiss Banking Ombudsman, 2023) amongst others. What is more, the recent introduction of so-called deepfake voices, by which novel speech utterances can be generated with an individual’s voice using deep neural network learning techniques, has led to crimes whereby victims are tricked to believe that one of their family members or close friends is in urgent need of financial help (Brewster, 2021; Flitter & Cowley, 2023; Khatsenkova, 2023). In such scenarios, a fraudsters’ age appearing in the context of the call and the victims’ age may differ drastically when an old person is frauded by a young caller, for example. Given these circumstances, we expect a strong increase in court cases, in which listeners appear as earwitnesses and are asked to give evidence whether the voice of a suspect is the voice of the person they spoke to on the phone during the frauded phone calls or otherwise give evidence in a formal voice identification procedure commonly known as a voice parade (McDougall, 2021; Robson, 2017). Such scenarios increase the diversity of person characteristics in interaction which may all have an impact on auditory speaker recognition and discrimination ability. Thus, a witness at court might have to judge whether the voice of a suspect from a strongly different age group belongs to a speaker they spoke to on the telephone during the voice crime. Listeners might have more experience with the voices of their own-age group due to relatively increased exposure compared to voices of other ages. Therefore, we asked whether listeners’ age group positively impacts discrimination of voices from the same age group, henceforth the own-age advantage in voice discrimination.

Own-group advantages (sometimes also known as own-group biases^1^) refer to a wide spectrum of phenomena by which an individual has an advantage in making perceptual judgements about a person based on some shared group attribution (in-group) compared to missing group attributions (out-group). These effects have dominantly been researched in the field of face identity processing (Denkinger & Kinn, 2018; Herlitz & Lovén, 2013; Mason, 1986; Meissner & Brigham, 2001; Rhodes & Anastasi, 2012; Sporer, 2001; Wright & Sladden, 2003). The predominant explanation of the effect suggests that observers have more experience with individual fine details of the own-group stimuli, which increases their recognition ability (Rhodes & Anastasi, 2012). Perhaps the best known example is the so-called own-race bias (Meissner & Brigham, 2001) by which faces of the perceivers own-ethnic group are better recognized compared to faces of a different ethnic group (often wrongly referred to as ‘race’). However, studies on own-age recognition advantages have generated conflicting results: while a number of studies reported superior sensitivity for faces of observers’ own-age group (Anastasi & Rhodes, 2005; Denkinger & Kinn, 2018; He et al., 2011; Wright & Stroud, 2002), other studies failed to observe it (Memon et al., 2003; Proietti et al., 2019; Rose et al., 2005). Further, some studies report the own-age recognition advantage in all of the investigated age groups (Wright & Stroud, 2002), while others observe it only for particular age groups (Anastasi & Rhodes, 2005; Denkinger & Kinn, 2018). It is worth noting that many tests used to assess face identity processing involve skewed sex and age compositions — in terms of either stimuli or participants (e.g. cf. Fysh et al. (2020); Stacchi et al. (2020)) — and are characterized by low to modest reliability in neurotypical observers (Bobak et al., 2023).

Own-group effects in *voice* identity processing received much less attention compared to factors influencing overall voice recognition performance discussed earlier. For example, it has been shown that listeners are better at describing accents which are geographically closer to their own (Braber et al., 2023; Tompkinson & Watt, 2018) and recognizing own-accent voices better than other-accent voices (Stevenage et al., 2012). Some studies demonstrated own-gender effects, whereby listeners were better at identifying voices of their own compared to other sexes (Roebuck & Wilding, 1993; Skuk & Schweinberger, 2013). Results on own-group effects in relation to speakers’ and listeners’ age are very limited. (Moyse et al., 2014) studied age estimation from voices and reported an own-group age estimation advantage for older adult listeners but not for younger adult listeners. Importantly, own-age advantages for voices (i.e., pertaining to voice recognition and discrimination) remain obscure.

Voice crime in the past mostly involved young male speakers aged approximately 18 to 40 years (Michael Jessen, German Federal Police Office, personal communication). Female voices appear less often as evidence in crime, even though their numbers have increased over the past 15 years and currently constitute between 5 and 10% of cases in the UK and Germany (Kirsty McDougall, University of Cambridge, personal communication; Richard Rhodes, The Forensic Voice Centre, personal communication; Michael Jessen, German Federal Police Office, personal communication). Cases like the ‘fake police officer’ possibly introduce a new dimension of female voice crime as female voices may intuitively be more trustworthy (Schirmer et al., 2020) when convincing older adult targets of crime in opening the door to an alleged police officer. As such, possible own-age voice discrimination advantages cannot be studied without considering own-sex effects.

In this study, we tested own-age effects in voice discrimination for male and female voices in male and female listeners using speaker discrimination task (i.e., same-/different-speaker judgements). We used speaker discrimination to avoid speaker- and listener-related familiarization effects. Listeners’ ability to learn and recognize voices can be affected by many factors, including set size (Legge et al., 1984), distinctiveness (Papcun et al., 1989), listeners cognitive abilities (Best et al., 2018) and familiarity (Case et al., 2018; Hollien et al., 1982). To limit these complex and compounding effects, we opted for a same/different judgement (i.e., voice discrimination) task, which does not require listeners to form and consolidate abstract voice representations in memory. We applied signal detection theory (SDT) to quantify listeners’ sensitivity (*d*′) as a measure of discrimination performance and response bias (*c*) as a measure of listeners’ tendency to respond ‘same’ or ‘different’, when the stimulus is ambiguous to them, for example. Response bias has been overlooked in the past and is particularly crucial for forensic applications because it indicates listeners’ tendencies to accept or reject a stimulus as familiar when in doubt, when stimulus quality is poor or in extreme cases without the presence of the stimulus itself.

## Material and methods

### Database and speakers

The materials for the current experiment were drawn from the TEVOID corpus (Dellwo et al., 2012; Pellegrino et al., 2021), which contains read and spontaneous sentence recordings from younger adults (henceforth YA speakers, range_Age_ = 18–32 years, *M*_Age_ = 30.3 years, standard deviation (SD) = 6.6 years) and older adults (henceforth OA speakers, range_Age_ = 66–81 years, *M*_Age_ = 71.7 years; SD = 4.9 years). All speakers were fluent native speakers of Zurich German, the Alemannic dialect spoken in the city and in most parts of the Canton of Zurich, and all sentence recordings were produced in Zurich German. The recordings were made in a sound treated booth using professional equipment and digitized at 44.1 kHz, 16 kbit/s bitrate. In this study, speech from 20 TEVOID speakers was included: 10 YA speakers (5 males and 5 females) and 10 OA speakers (5 males and 5 females).

### Listeners

In total, 74 listeners completed the experiment, including 42 YA listeners (21 males, range_Age_ = 19–35 years, *M*_Age_ = 26.7 years; *SD*_Age_ = 3.6 years) and 32 OA listeners (14 males, range_Age_ = 65–83 years, *M*_Age_ = 73 years; *SD*_Age_ = 5.7 years). All listeners were native speakers of Swiss German and had lived in Zurich for a substantial number of years. They did not learn a second language before the age of seven. None of the listeners reported any history of speech or language deficits.

To ensure that OA listeners’ performance was not influenced by age-related cognitive impairment, we performed Montreal Cognitive Assessment (MoCA) (Nasreddine et al., 2005). 30 OA listeners had MoCA score ≥ 26 suggesting they had no cognitive impairment, while the MoCA data from two OA listeners were not recorded due to technical reasons. Hearing loss in OA listeners did not exceed moderate hearing loss: mean pure-tone audiometry (PTA) threshold was 19.5 dB, standard deviation 11.3 dB for the octave frequencies from 0.5 to 4 kHz. Listeners with > 50 dB hearing threshold in the better hearing ear were excluded, since this is the upper threshold for moderate hearing loss defined by WHO (World Health Organization, 2021). All OA listeners had symmetrical hearing (< 15 dB interaural threshold difference), and none of them were usering hearing aids. No PTA thresholds were measured for YA listeners.

### Materials

We used a speaker discrimination task (same/different judgement) to investigate speaker discrimination performance in YA and OA listeners. To create same- and different-speaker pairs, we used read sentence recordings from the TEVOID corpus (see Sect. “Database and speakers’). In total, the pool of 1820 read sentence recordings was used to construct stimuli pairs (20 speakers × 91 sentences). To create speaker pairs, sentence stimuli were resampled to 10 kHz, and 800 ms snippets were extracted from each sentence midpoint using Hanning window over the frequency range of 80–5000 Hz with 40 Hz slope. Each speaker pair thus consisted of two 800 ms speech snippets separated by a 500 ms silent interval. While extracting snippets from a sentence midpoint may lead to a decrease in grammaticality and intelligibility, multiple studies show that voice recognition and discrimination is possible with unintelligible stimuli, for example, in time-reversed or noise-vocoded speech (Fleming et al., 2014; Garrido et al., 2009). Furthermore, we chose 800 ms snippet length for the current task since previous studies show that voice discrimination performance is optimal with stimuli length between 500 and 1000 ms and that further increase in duration does not increase the performance (Bricker & Pruzansky, 1966; Pollack et al., 1954). We limited the bandwidth of our stimuli to exclude any possible high-frequency artefacts that might be audible, and to present listeners exclusively with relevant speech and speaker information below 5 kHz. The remaining information contains sufficient speaker-specific voice detail for successful discrimination and recognition, as demonstrated exhaustively by studies using landline telephone speech as stimuli which has bandwidth of approximately 300–3400 kHz (Köster & Schiller, 1997; McDougall, 2021; Nolan et al., 2013; Rathborn et al., 1981; Schiller & Koster, 1996).

Each listener received a unique subset of 80 stimuli pairs, in which equal number of same- and different-speaker pairs, younger and older speaker pairs, as well as female and male speaker pairs were included. In different-speaker pairs, stimuli were always matched for age and sex, so no speaker pairs contained mismatched stimuli by age and/or sex of the speakers. Sentence numbers within each speaker pair were mismatched. This way, in same-speaker pairs listeners never compared two identical stimuli tokens. In different-speaker pairs, listeners never compared linguistically identical sentences produced by two different speakers. Speaker pairs were created using Praat scripts, Praat version 6.1.51 (Boersma & Weenink, 2025).

### Procedure

Testing took place in person at the Linguistic Research Infrastructure (LiRI) laboratory at the University of Zurich. Listeners performed the task in a soundproof booth, where they were seated at a desktop PC. Sound was played back through a loudspeaker, since not all OAs were comfortable with wearing headphones and since such listening conditions may be considered more realistic compared to listening to voices via closed-up headphones. The loudspeaker was situated to the left side of the PC monitor, at approximately 70 cm distance from the listeners, and they could adjust the loudness level to their comfort. The experiment was created and administered via the Gorilla experiment builder (Anwyl-Irvine et al., 2020). On every trial (*N* = 80), listeners heard a pair of audio snippets and were instructed to indicate whether both snippets stemmed from one speaker or from two different speakers using buttons on the screen. No other answer options were available. The audio was played automatically, and listeners heard stimuli in each trial only once before giving an answer to ensure that all listeners receive the same duration of voice input. Listeners were instructed to complete the task at their own pace, and no time limits were introduced for completing the task (it took on average 10 min to complete). Eight attention checks were also included in the task to ensure listeners stayed attentive during the experiment. During attention checks, listeners were shown animal cartoon pictures on the screen and instructed to type animals’ names in the text field below.

### Measures

Using signal detection theory (SDT), listeners’ performance was quantified with measures of sensitivity (*d*′) and response bias (*c*) (Macmillan & Creelman, 2004; Stanislaw & Todorov, 1999). Sensitivity is broadly conceived as the ability to perceive a signal (Macmillan & Creelman, 1991), whereas response bias is defined as subjects’ tendency to prefer one type of response over the other (Stanislaw & Todorov, 1999). *d*′ and *c* values were calculated per listener and condition, such that from each listener we obtained four *d*′ and four *c* values corresponding to each of the four experimental conditions (i.e., YA and OA speakers, as well as female and male speakers). Data were inspected for quality prior to performing statistical analyses. Data would have been excluded if 20% or more of attention checks were solved incorrectly and/or if performance in the discrimination task was at chance level or below (i.e., 50% or less correct responses), since this might indicate that listeners did not stay attentive or were unable to solve the task, for example, because of an inability to recognize voices. All listeners completed all attention checks correctly and performed significantly above chance level, so no data were discarded based on these exclusion criteria. Thus, the final dataset for analyses comprised 296 *d*′ and 296 *c* values (74 listeners × 4 conditions).

### Acoustic analyses

**Fig. 1:**
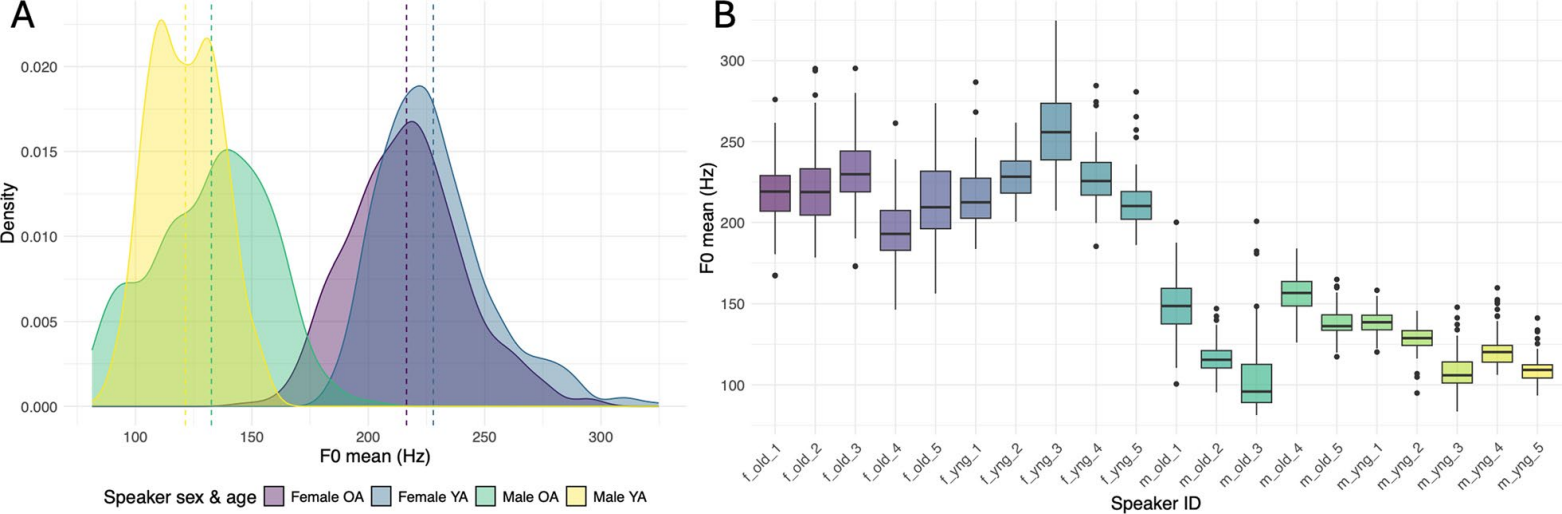
**A** Density plots showing distributions of mean f_0_ values for younger and older, as well as male and female speakers. Dashed lines represent group means. **B** Boxplots showing mean, range and interquartile range of mean f_0_ values per speaker. ‘f_old’—older female speakers, ‘f_yng’—younger female speakers, ‘m_old’—older male speakers, ‘m_yng’—younger male speakers. 1, 2, 3, 4, 5—individual speaker IDs

To inspect overall acoustic characteristics of our voice sample, we investigated acoustic differences between male and female, younger and older voices. We extracted *f*_0_ contours from all 800 ms speech snippets which were presented to the listeners and calculated mean *f*_0_, *f*_0_ range (*f*_0*max*_–*f*_0*min*_) and *f*_0_ coefficient of variation, a standardized measure of *f*_0_ variation computed as (*f*_0*SD*_*/f*_0*Mean*_)*100. All measures were calculated on a linear scale in Hz in Praat version 6.1.51 (Boersma & Weenink, 2025) using gender-specific pitch ranges (75–400 Hz for male, 120–600 Hz for female voices). Figure 1B presents distributions of individual speaker’s *f*_0_ mean values in Hz.

*f*_0_ mean, *f*_0_ range and *f*_0_ coefficient of variation were modelled in three separate mixed-effects models (Baayen et al., 2008) using *lmerTest* package in R (Kuznetsova et al., 2017). All models had identical structure: fixed effects for speaker sex (categorical with two levels: male and female) and age (categorical with two levels: OA and YA), as well as by-speaker and by-sentence random slopes for age and sex. This model had the maximal random effect structure justified by our experimental design, which should optimize generalization of the findings (Barr et al., 2013). Significance was assessed using *p*-values from the Satterthwaite approximation for degrees of freedom in the *lmerTest* package. Two-way interaction between main effects of speaker age and sex on *f*_0_ mean, *f*_0_ range and *f*_0_ coefficient of variation was not significant in neither of the three models (all *p* > 0.05). We therefore fitted all three models with fixed effects of speaker sex and age without interaction on *f*_0_ mean, *f*_0_ range and *f*_0_ coefficient of variation, respectively.

For the *f*_0_ mean model, only the effect of speaker sex was significant, whereby male speakers had significantly lower mean *f*_0_ compared to female speakers. (*β* = − 103.3, *SE* = 11.4, *z* = − 9.06, *p* < 0.0001), whereas speaker age effect was not significant (*β* = 3.1, *SE* = 5.2, *z* = 0.6, *p* > 0.05) (Fig. 1A). For *f*_0_ range model, both effects of speaker sex (*β* = 72.8, *SE* = 7.7, *z* = 9*.*4*, p* < 0.0001) and age (*β* = 24.6, *SE* = 5.3, *z* = 4*.*7*, p* = 0.004) were significant, whereby female speakers and older speakers had significantly larger *f*_0_ range compared to male speakers and younger speakers, respectively (Fig. 1A). Similarly, for *f*_0_ coefficient of variation model, both effects of speaker sex and age were significant, whereby female speakers (*β* = 2.8, *SE* = 0.7, *z* = 3*.*6*, p* = 0.009) and older speakers (*β* = 3.6, *SE* = 0.7, *z* = 5*.*2*, p* = 0.003) had significantly larger *f*_0_ range compared to male speakers and younger speakers, respectively (Fig. 1A).

As additional analysis, we converted *f*_0_ mean, range and coefficient of variation values to logHz and rerun all regression analyses with logHz values as dependent variables. The conclusion from regression analyses with logHz were in the same direction as when using values on a linear scale: (1) no significant interactions between speaker age and sex variables in neither of the models; (2) for the *f*_0_ mean model, only the effect of speaker sex was significant; and (3) for the *f*_0_ range and *f*_0_ coefficient of variation models, both the effects of speaker age and sex were significant.

## Results

**Fig. 2:**
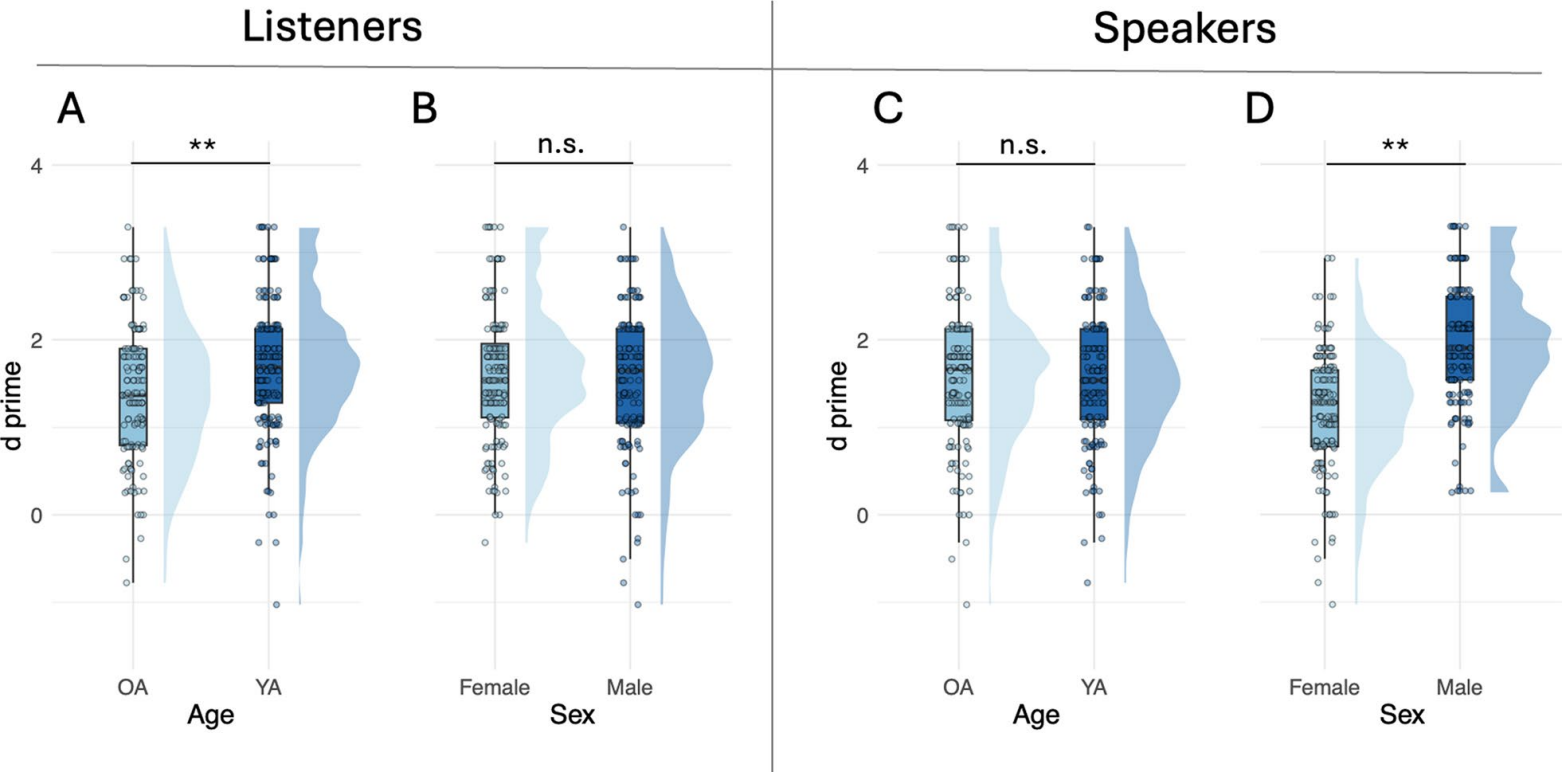
Raincloud plots showing distributions and boxplots with median, range and interquartile range of sensitivity (*d*′) values for: **A** listener age (younger and older listeners), **B** listener sex (male and female listeners), **C** speaker age (younger and older speakers) and **D** speaker sex (male and female speakers). The shaded region in each plot shows the distribution of the data. Data from individual participants are indicated by transparent dots. YA = younger adults; OA = older adults. Significance codes: 0 ‘***’ 0.001 ‘**’ 0.01 ‘*’ 0.05 ‘.’ 0.1 ‘’ 1, ‘n.s.’ not significant

**Fig. 3:**
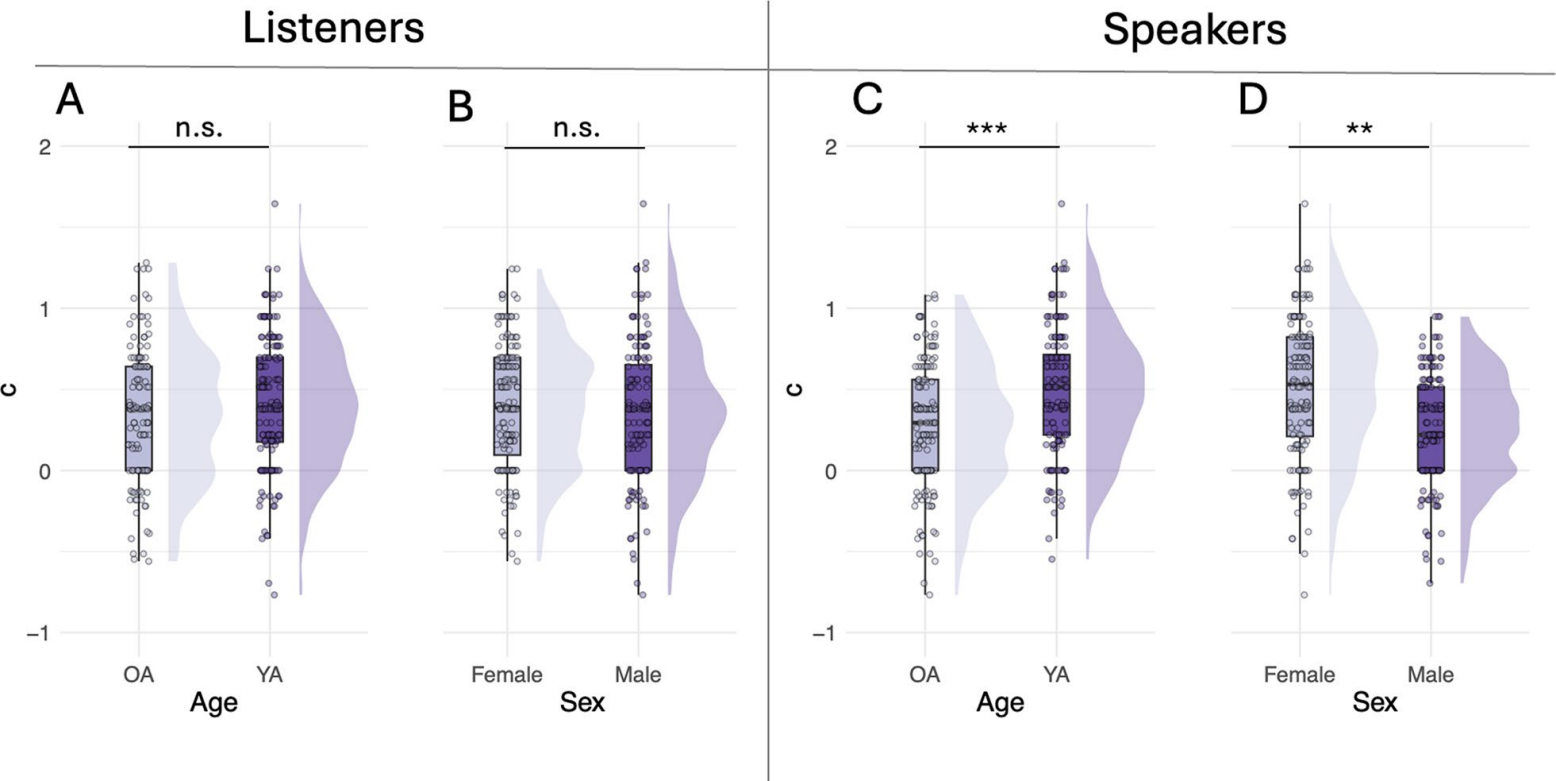
Raincloud plots showing distributions and boxplots with median, range and interquartile range of response bias (c) values for: **A** listener age (younger and older listeners), **B** listener sex (male and female listeners), **C** speaker age (younger and older speakers) and **D** speaker sex (male and female speakers). The shaded region in each plot shows the distribution of the data. Data from individual participants are indicated by transparent dots. YA = younger adults; OA = older adults. Significance codes: 0 ‘***’ 0.001 ‘**’ 0.01 ‘*’ 0.05 ‘.’ 0.1 ‘’ 1, ‘n.s.’ not significant

All statistical analyses were conducted in R version 4.0.3 (R Core Team, 2024). To assess the effects of listener age, listener sex, speaker age and speaker sex on *d*′ and *c*, we used two four-way mixed ANOVAs (one for *d*′ and one for *c*, respectively) which test for all main effects and interactions between our four factors of interest: listener age (YA and OA listeners), listener sex (male and female), speaker age (YA and OA speakers) and speaker sex (male and female) on *d*′ and *c*.^2^ Four-way interactions were not significant for neither *d*′ nor *c*. Also, no interactions which included listener sex factor were significant, and listener sex was also not significant as a main effect neither for *d*′ nor for *c* (Figs. 2B and 3B, respectively). Therefore, we collapse further results across male and female listeners and examine the interactions between the remaining three factors (i.e., listener age, speaker age and speaker sex) and their main effects on *d*′ and *c*. We used the three-way robust mixed ANOVAs which tests for all main effects and interactions using trimmed means (Sect. 7.1 in (Wilcox, 2021)). Robust approaches offer higher statistical power and robustness to deviations from the assumed optimal distribution parameters, as suggested by previous studies with the experimental design similar to ours (Ramon, 2015; Ramon et al., 2011). Trimmed means is a robust approach typically used to minimize the standard error of the data containing outliers and small deviations from normality (Wilcox & Keselman, 2003). It is especially suitable for designs with unequal sample sizes, since one of its advantages is to deal with the unequal variances of the involved samples (Mair & Wilcox, 2020). The three-way robust ANOVAs for *d*′ and *c* were fitted using the function *t*3*way* from the *WRS*2 package (Mair & Wilcox, 2020).

Following Mair and Wilcox (2020), effect sizes for the main effects for independent samples (i.e., listener age) were calculated using AKP-type effect sizes (*d*_*t*_), a robust alternative to Cohen’s *d* using the function *akp.effect* from the *WRS*2 package (Mair & Wilcox, 2020). AKP-type effect sizes of 0.2, 0.5 and 0.8 correspond to small, medium and large effect sizes, respectively (Wilcox, 2021). As suggested by Mair and Wilcox (2020), effect sizes for main effects for dependent samples (i.e., speaker age and speaker sex) were produced using Yuen’s trimmed mean *t* test for dependent samples calculated with function *yuend* from the *MASS* package (Venables & Ripley, 2002). It reports the explanatory measure of effect size (*ξ*), which is interpreted as follows: values of 0.15, 0.35 and 0.50 correspond to small, medium and large effect sizes, respectively (Sect. 5.3.4 in Wilcox (2021)). Below we report the results for *d*′ and* c* in detail.

### Sensitivity (*d*′)

The results of the robust three-way ANOVA on *d*′ showed no significant three-way or two-way interactions between listener age, speaker age and speaker sex (all *p* > 0.05). The main effect of listener age was significant (*F*(1, 72) = 11.4, *p* < 0.001, *d*_*t*_ = 0.57), whereby YA listeners performed significantly better compared to OA listeners (Fig. 2A). However, the main effect of speaker age was not significant (*F*(1, 72) = 0.2, *p* = 0.7, *ξ* = 0.01) indicating that there was no significant difference between discrimination scores for YA and OA speakers (Fig. 2C). Lastly, the main effect of speaker sex was also significant (*F*(1, 72) = 76.6, *p* < 0.001, *ξ* = 0.79), whereby male speakers were discriminated significantly better than female speakers (Fig. 2D).

### Response bias (*c*)

Similar to *d*′, a three-way robust ANOVA on *c* showed no statistically significant three-way or two-way interactions between listener age, speaker age and speaker sex on *c* (all *p* < 0.05). Likewise, the main effect of listener age was not significant (*F*(1, 72) = 1.2, *p* = 0.3, *d*_*t*_ = 0.22) suggesting the two listener groups did not differ significantly in terms of response bias (Fig. 3A). However, the main effect of speaker age was significant (*F*(1, 72) = 18.4, *p* < 0.0001, *ξ* = 0.47), whereby listeners were significantly more biased towards responding ‘same’ when hearing YA speaker pairs compared to OA speaker pairs (Fig. 3C). Lastly, the main effect of speaker sex was also significant (*F*(1, 72) = 24.3, *p* < 0.001, *ξ* = 0.53) suggesting that listeners were significantly more biased towards responding ‘same’ when hearing female speaker pairs compared to male speaker pairs (Fig. 3D). It should be noted that response bias values in all experimental conditions were significantly shifted above zero (assessed with one-sample t-tests, all *p* < 0.05), which could be a result of the experimental design, namely, short stimulus duration and the overall nature of a discrimination task (see Discussion). Crucially, however, the differences between YA and OA speakers, as well as between male and female speakers were significant.

## Discussion

This study investigated own-age effects in voice discrimination. While the study did not find a performance difference in terms of a higher discrimination accuracy for the own-age group stimuli, we crucially discovered a listener bias revealing a preference for ‘same’ response in case of younger speakers and female speakers. It is possible that such bias can be accounted for by acoustic differences between male and female speakers. Males typically exhibit comparatively lower fundamental frequencies than females—a reflection of both physiological and cultural factors (Munson & Babel, 2019). Since the harmonic components of the glottis signal are simple multiples of *f*_0_, the harmonic signal in male speakers is much tighter compared to female speakers (Simpson, 2009). It is unclear, however, whether this sparser sampling of harmonics in female voices (Munson & Babel, 2019) leads to less vocal tract individualities being revealed in spectral envelopes of female voices. It furthermore remains to be investigated if narrower spacing between harmonics in males is advantageous for voice identity recognition. Superior recognition performance for male compared to female voices has been previously reported for x-vector and i-vector based automatic speaker recognition systems in balanced male and female datasets (Kathiresan, 2021). However, human listeners and machines use different features to process voices (Park et al., 2018), and their performance is tested with different tasks. Future studies could systematically test the relationship between vocal tract sampling and recognition performance. One possibility would be to extract speakers’ spectral envelopes and fill them with harmonic signal of different densities while leaving the spectral envelope unchanged. Different listener groups can then be trained to remember voice identities either by listening to spectrally dense stimuli or to spectrally undersampled stimuli. Afterwards, listeners will perform a voice recognition test, which can elucidate whether a spectrally dense signal is beneficial for learning and recognizing voice identities. In addition, future studies could address whether female voices are perceived as more similar by the listeners compared to male voices. If confirmed, such effect could imply that listeners develop a bias by experience in the sense that whenever the stimulus is ambiguous (or when the stimulus quality is poor), the choice is on ‘same’ rather than on ‘different’ response.

In addition, studies show that female speakers have wider *f*_0_ range and variability than male speakers (Haan & van Heuven, 1999; Traunmüller & Eriksson, 1995), and acoustic analyses of our stimuli confirm that. It is possible that increased *f*_0_ range and variability make it more challenging for the listeners to ‘tell speakers together’ (Lavan et al., 2019a, 2019b; Lavan et al., 2019a, 2019b), i.e., generalize over highly variable samples and correctly attribute them to the same-speaker identity. This, in turn, might lead to a decreased discrimination accuracy of female compared to male voices. Another source for higher similarities in female voices might be the fact that they show a higher phonetic convergence compared to males, i.e., they change their vocal parameters to sound more similar to their interlocutors (Namy et al., 2002). This may be an additional factor that contributes to female voices being on the whole more similar than male voices and thus fostering a bias by experience. In other words, because listeners are already unconsciously aware of the tendency of female speakers to sound more similar to the interlocutors compared to male speakers, this may contribute to forming a perceptual bias that female speakers are per se more similar compared to male speakers. For a comprehensive review of various acoustic, linguistic and social factors contributing to variation in male and female speech, see (Babel & Munson, 2014; Munson & Babel, 2019).

A factor that may contribute to the same-speaker bias in younger voices may possibly lie in older speakers being experienced as more different in everyday situations due to their development of a variety of source signal individualities in terms of laryngealizations over the years that are not yet present in younger speakers. Age-related non-pathological changes in voice include changes to fundamental frequency (*f*_0_), increased *f*_0_ variability patterns within speakers (as confirmed by acoustic analyses of our stimuli), decreased harmonic to noise ratio, increased jitter and shimmer, as well as decreased acoustic intensity (for a review, see Goy et al. (2016) and Schultz et al. (2023)). Such age-related voice changes may contribute to a bias that younger voices — not showing these distinctions — are per se more similar. In other words, younger voices might be perceived as being more similar in the presence of more variable voices of the older speakers.

Voice perception relies on many different features beyond *f*_0_. While detailed acoustic analysis of the stimuli is beyond the scope of this paper, future studies could investigate in detail the relationship between, for example, differences in mean *f*_0_ or *f*_0_ variation between speakers in a pair and listeners’ responses. Such item-wise analysis could clarify whether pairs of speakers with large differences in *f*_0_ or *f*_0_ variation would be easier to discriminate than pairs in which speakers’ *f*_0_ is more similar. Such findings might suggest that it is not speaker sex per se that is driving discrimination differences in sensitivity and bias, but rather speakers’ *f*_0_ properties. If speakers have distinct *f*_0_ mean and smaller *f*_0_ variance, as is the case for male speakers, it is possible that speaker pairs will be well discriminated by pitch alone. On the other hand, if female speakers in the sample have higher *f*_0_ variance, then *f*_0_ mean would be a less reliable cue for voice discrimination: the same female speaker might have very different pitch values across different speech samples, while two different female speakers might have very similar mean *f*_0_ values (i.e., if there is high overlap in their *f*_0_ ranges). These hypotheses remain to be addressed by future research.

Note that our participants generally adopted a loose response criterion, as evidenced in the more frequent ‘same-speaker’ response bias across all conditions (Fig. 3). Crucially, however, the differences between experimental condition in response bias were significant. Shorter stimuli offer the listener less detail to be compared and thus to arrive at a ‘different-speaker’ response, thus, it is plausible that the number of false ‘same-speaker’ responses should increase. Previous research shows that voice discrimination as compared to voice recognition tasks are associated with higher ‘same’ response biases. Kreiman and Papcun (1991) directly compared voice recognition and discrimination performance and found that listeners were overall more biased towards ‘same’ response in discrimination task, but not in recognition task. The authors hypothesized that both stimulus duration (they used shorter stimuli in discrimination compared to recognition task) and task demands causing a shift in response criteria could account for these findings. Both across Kreiman and Papcun (1991) and our study, listeners never compared two identical stimuli is same-speaker pairs or two sentences with the same content in different-speaker pairs. Thus, linguistic content between stimulus 1 and stimulus 2 in each speaker pair was different. This means listeners had to accept a certain amount of difference between stimuli as possibly belonging to a same-speaker identity, because speakers sound different when producing different linguistic structures and even when producing the same linguistic content multiple times (Kreiman & Papcun, 1991).

Other findings of this study also brought important phenomena to light. In terms of sensitivity (*d*′), we found no interactions between listener and speaker age, which would have suggested the presence of an own-age discrimination advantage. This contrasts with the often reported own-age advantage for face identity processing (Denkinger & Kinn, 2018; Rhodes & Anastasi, 2012; Wright & Stroud, 2002), which might point towards differential processing of faces and voices, as suggested by previous research: for example, faces may provide more reliable identity information than voices (Brédart et al., 2009). Also, identity and sex information might be processed separately for faces, but not for voices (Burton & Bonner, 2004). Therefore, it is possible that an own-age recognition advantage for facial identity could follow different principles than those involved in voice processing. However, future studies should disambiguate an own-age processing advantage with different listener populations (e.g. children and middle-aged adults), languages and listening conditions (e.g. speech in noise).

Our results also showed that YA listeners outperformed OA listeners in terms of sensitivity (*d*′), which is in line with previous literature about the listener age effect on various voice perception tasks (Best et al., 2018; Clifford, 1980; Kausler & Puckett, 1981; Yonan & Sommers, 2000; Zaltz & Kishon-Rabin, 2022). This might be expected given a general age-related decline in hearing and cognitive functions in older adults (Deary et al., 2009). A novel finding was observed regarding a speaker age effect, whereby listeners could discriminate YA and OA speakers equally well. Previous research shows that accurate speaker discrimination and recognition helps listeners to structure and process linguistic content of speech (Kreiman & Sidtis, 2011; Nygaard & Pisoni, 1998), therefore, it is equally important for listeners to accurately discriminate speakers of different ages to accurately process and understand speech.

As for the effects of listener and speaker sex on sensitivity, we found no significant interaction between these factors on *d*′, which contrast with studies reporting an own-sex voice recognition advantage (Roebuck & Wilding, 1993; Skuk & Schweinberger, 2013; Wilding & Cook, 2000). However, previous findings of these interactions appear inconsistent, with the own-sex advantage either present in both male and female listeners (Roebuck & Wilding, 1993), or confined to only female (Wilding & Cook, 2000) or male (Skuk & Schweinberger, 2013) listeners. Instead, our results showed that male speakers were discriminated better than female speakers, which aligns with the results reported by Best et al. (2018) and Thompson (1985), as well as corroborates results from the automatic speaker recognition domain showing a consistent performance advantage for male compared to female speakers (Kathiresan, 2021). Male voices tend to have lower *f*_0_ compared to female voices as a result of longer vocal folds in men compared to women (Puts et al., 2016). Therefore, it is possible that male voices are easier to discriminate due to a denser spectrum of harmonics that better samples the individual vocal tract characteristics (Dellwo et al., 2018). On the other hand, male and female *listeners* did not differ in terms of sensitivity (*d*′), which is in line with previous literature (Clifford, 1980; Thompson, 1985; Yarmey & Matthys, 1992; Yarmey et al., 2001). Only the early work by McGehee (McGehee, 1937) showed that male listeners outperformed female listeners using a large sample of graduate students. However, our study included both younger and older adult listeners, which could explain differences in our results compared to those of McGehee (1937).

Evidence from the field of face identity processing suggests that own-age advantage can be explained by the more extensive exposure to the faces of observers’ own-age group, which aids successful recognition (Rhodes & Anastasi, 2012). On the other hand, social–cognitive theories suggest that superior recognition for in-group faces is driven by an initial categorization of a face as belonging to an in-group (Rhodes & Anastasi, 2012). Categorizing a face as an in-group one aids an observer in successful encoding of face individual properties, which then facilitate recognition (Hugenberg et al., 2010). By contrast, if a face is categorized as an out-group one, the subsequent encoding focuses on the category-level, rather than individual-level features (Levin, 2000).

In voice identity processing, it could be that own-age effects will be amplified by the *explicit* training and experience with the voices of the own-age group. In everyday situations, we typically acquire voice information implicitly, without consciously attending to it, therefore, some indexical cues might remain unattended to by the listeners. On the other hand, if listeners are instructed to actively memorize individual properties of voices of their own-age group, the own-age effects might be detectable. Our procedure did not involve any training or prior exposure to voices, but this can be addressed by future studies using voice recognition/identification tasks. Voice discrimination and recognition are distinct but related abilities supported by partially dissociated cognitive processes and response strategies: while identifying familiar voices involves holistic pattern recognition and matching it to a name or a person, discriminating unfamiliar voices relies on feature analysis and comparison of basic acoustic parameters between the compared voices (Maguinness et al., 2018; Van Lancker & Kreiman, 1987). Such a partial dissociation between voice discrimination and recognition is further supported by evidence from brain-lesioned patients with impaired ability to discriminate voices but intact ability to recognize familiar voices and vice versa (Maguinness et al., 2018; Van Lancker et al., 1988). Therefore, it is possible that a different pattern of results would emerge in terms of own-age advantages when using a recognition instead of discrimination task. Also, as discussed above, differences can be expected in response bias, especially if voice recognition task involves open set design (i.e., listeners are informed that a target speaker may or may not be in the recognition set), in which case listeners may adopt an overall stricter response criterion (see discussion in Kreiman and Papcun (1991)).

To summarize, this study examined own-age effects in voice discrimination using measures of sensitivity (*d*′) and response bias (*c*). Our results indicated that YA listeners discriminate speakers better than OA listeners and that male speakers were discriminated better than female speakers. We also showed that listeners are significantly biased towards responding ‘same’ when hearing young speaker pairs and female speaker pairs. These results might also be relevant for a variety of forensic procedures involving voice evidence. First, our stimuli had limited bandwidth (80–5000 Hz) and thus resembled realistic casework audio material better than studio-quality stimuli often used in speaker recognition tasks. Furthermore, it is possible that in voice crime cases involving young speakers and female speakers it might be more challenging for the earwitnesses to tell apart younger and female voices, especially when the stimulus quality is poor and exposure to the voice is brief, which is often the case for forensic voice evidence. Previous work shows that the risk of bias increases with decreasing stimulus quality (Forensic Science Regulator, 2020), and it is essential that forensic voice experts take into account all possible sources of bias when assessing an earwitness’ testimony in court. This is especially relevant given that most of voice crime cases involve young speakers and that the number of voice crime cases with female speakers has increased in recent years, especially in the financial crime domain and in ‘fake police officer’ crimes discussed in Introduction.

Even though our experiment design differs from the typical voice parade procedure, the observed findings are of interest for forensic experts when constructing voice parades for earwitness identification testimonies in court. In a voice parade, an earwitness is asked to identify the voice of the speaker they heard at the crime scene from a collection of recorded speech samples by a suspect and a number of foils (McDougall, 2021). Voice parades are prepared using thorough experimental procedures: typically, a sample of a suspect’s speech is compiled from excerpts extracted from a recorded police interview, and speech samples from foil speakers are constructed from recordings of similar quality, speaking style and duration (McDougall, 2021). The foil speech samples must be screened to ensure that foil speakers do not stand out in terms of accent, pitch and speaking rate compared to the suspect’s voice (Home Office, 2003). In other words, voice parade procedures require a rigorous phonetic screening to select suitable foil speakers. Thus, research evidence obtained under controlled laboratory conditions is important for daily forensic investigations related to formal voice identification procedures.

In addition, future research might investigate whether forensic voice experts are also liable to such biases for younger voices and/or female voices, since this would have an impact on forensic phonetic casework on a much broader level. However, (Bartle & Dellwo, 2015) showed that voice experts tend to have a conservative bias when unsure, meaning that they tended to respond that samples come from different speakers. Thus, expert listeners should be less liable to a positive response bias as compared to lay listeners. Also, expert listeners should also be aware of the range of cognitive biases which may affect recognition performance and use some strategies to mitigate the influence of biases (Gold & French, 2019; Rhodes, 2014). Further, the analysis carried out by the expert listeners in forensic speaker comparison is categorically different from ad hoc same/different judgments in our experiment. Forensic voice experts systematically use a wide range of analytic methods and take decisions after applying typically complex acoustic, auditory and automatic procedures.

At least two components contribute to the perception of person similarity: the signal itself (i.e., the acoustic distinguishability of voices) and the perceived distance between voices (i.e., perceptual factors impacting whether voices are perceived as more or less different). Recently, researchers became increasingly interested in equating acoustic differences between voices with their perceived differences for human listeners. State-of-the-art deep neural networks, for example, produce vectors from acoustic voice samples that allow maximum classification and recognition accuracy. However, to what degree human perceptual judgements align with distances produced by neural networks is unclear. Despite the increased awareness in acoustic and perceptual factors, they are often viewed as somehow mechanically contributing to voice recognition. Current models of voice perception suggest that voices are represented in terms of their acoustic deviation from a voice prototype conceptualized as average of all voices heard by a listener (Belin et al., 2011; Latinus et al., 2013; Lavner et al., 2001; Maguinness et al., 2018). Our results highlight that voices may be judged as more or less similar based on characteristics other than stimulus acoustics and demonstrate that cognitive biases can be an important component of voice perception. However, cognitive biases have not been paid much attention to in the past and are not accounted for by current voice perception models. It would thus be interesting to explore further their origin and the impact they have on recognition accuracy and perception of other person characteristics.

It also seems plausible that there are evolutionary mechanisms employed that lead to listeners being biased towards perceiving some groups of listeners with higher similarity than others. In the case of younger speakers this might be rooted in mechanisms by which younger individuals are possibly more viewed as part of a group and not as individuals to the same degree as older adults. This can be addressed by future studies.

## Data Availability

Due to the data protection guidelines of the University of Zurich, the raw audio recordings used during this study are not publicly available. However, mel-frequency cepstral coefficients (MFCCs) of the audio recordings are available for scientific purposes from the first author upon request. Behavioural data and analyses code are publicly available in the study's Open Science Framework repository: https://osf.io/sg96w/

## Footnotes

1 We refrain from using the term ‘bias’ for this effect since it is ambiguous: it can either mean a recognition advantage for own-group stimuli (i.e., so-called performance bias) or a response bias, whereby individuals tend to choose one of the response options in the experiment significantly more.

2 Next to the chosen statistical procedure, we considered numerous other plausible models. One of them was linear regression. However, we found that this procedure was not suitable for our data since visual inspection revealed outliers. Another procedure, linear mixed effect regression, was also not applicable since single-trial data were collapsed during calculations of d′ and c measures. Also, nonparametric alternatives to ANOVA such as Kruskal–Wallis or Friedman’s tests were not suitable for our experimental design since they do not allow to test for interactions. Lastly, there appears to be no robust alternative for the four-way mixed ANOVAs in R, which would mitigate the presence of outliers. This is why we tested the interactions and main effects of our four factors with normal four-way mixed ANOVAs.
